# The effect of gender in binge eating behavior in Chinese culture: the serial mediation model of body dissatisfaction and self-acceptance

**DOI:** 10.3389/fpsyg.2023.1285272

**Published:** 2023-12-08

**Authors:** Chunlu Li, Shuhui Lyu, Jimin Yan, Xiaolu Meng

**Affiliations:** ^1^Department of Histology and Embryology, School of Basic Medical Sciences, Guizhou Medical University, Guiyang, Guizhou, China; ^2^Key Laboratory for Research on Autoimmune Diseases of Higher Education Schools in Guizhou Province, Guiyang, China; ^3^Guizhou Health Development Research Center, Guiyang, China; ^4^Department of Psychology, School of Medical Humanitarians, Guizhou Medical University, Guiyang, China

**Keywords:** gender difference, body dissatisfaction, self-acceptance, binge eating behavior, social culture

## Abstract

**Introduction:**

The gender difference of binge eating behavior been highlighted by previous studies. However, psychological mechanisms underlying the gender difference of binge eating behavior remain unclear. This study addressed this issue from a sociocultural perspective.

**Methods:**

Firstly, we investigated the mediation effect of body dissatisfaction on the gender difference of binge eating behavior. Secondly, we examine the serial mediating role of body dissatisfaction and self-acceptance in gender differences of binge eating behavior. Here, we analyzed data from 703 Chinese university students using SPSS 26.0 and SPSS PROCESS.

**Results:**

In Chinese culture, body dissatisfaction and self-acceptance independently or through a serial way mediate the gender differences in binge eating behaviors.

**Discussion:**

We discussed the implications and limitations of the present study.

## Introduction

Binge eating behavior refers to a person eating much more than majority of the people under similar circumstances, often accompanied by a sense of loss of control. It is a common feature of several eating disorders, including binge eating disorder (BED), anorexia nervosa (AN) and bulimia nervosa (BN) (American Psychiatric Association, 2013; Peat et al., 2017). The life-time prevalence of binge eating disorder were 1.9% across 14 World Health Organization World Mental Health (WMH) countries (Kessler et al., 2013). Binge eating behaviors are associated with many health-related problems, such as weight gain or obesity (Herpertz-Dahlmann, 2015), depression and anxiety (Udo and Grilo, 2019), non-suicidal self-injury behaviors (Pak et al., 2021), as well as lower levels of happiness and quality of life (Pak et al., 2021). There is ample evidence to suggest that binge eating behavior is more prevalent in females. The lifetime prevalence estimates of BED among 14 WMH countries were 2.4% in females and 1.0% in males (Kessler et al., 2013), and 5.3% for females as well as 0.7% for males in Italy (Pace Serena and Muzi, 2019). In addition, one study found that 53.6% of girls reported engaging in at least one key eating disorder behavior, compared to 30.5% of boys in Australia (White et al., 2014). In China, there is also a significant gender difference in the occurrence of binge eating behavior. Based on a study conducted with 1,103 Chinese university students, it is estimated that the prevalence of binge eating behavior is three times higher in females compared to males (Yan et al., 2018). Given the serious consequences of binge eating, it’s important to understand why there are gender differences.

In the existing theoretical and empirical research, the gender differences of binge eating behavior have been examined from three perspectives: neurophysiological, cognitive, and sociocultural. Neurophysiological studies have provided evidence linking gonadal steroid hormones to the occurrence of binge eating behavior. For instance, estrogen is known to interact with various central and peripheral signals, influencing brain pathways that are involved in the motivational and rewarding aspects of eating behaviors (Richard et al., 2017; Rivera and Stincic, 2018; Breton et al., 2023). Both animal and human studies have shown that lower levels of estradiol were associated with higher levels of emotional eating and binge eating frequency (Klump et al., 2013, 2018). Moreover, neuroimaging studies and non-invasive neuromodulation approaches have revealed that males and females differ in neural mechanisms and brain activation underlying food choice. Animal studies have observed differential responses to food cues in the orbitofrontal cortex and hippocampal-amygdala system between female and male rodents (Anderson and Petrovich, 2017, 2018; Anderson and Petrovich, 2018). Research focusing on adults has indicated that compared with males, females often exhibit greater activation in brain regions associated with sensory processing, executive control and inhibition, as well as reward functions, following exposure to visual and gustatory stimuli (Culbert et al., 2021).

Cognitive theories as a psychological perspective emphasize the role of negative automatic thoughts in relation to aspects of eating disorders. It has been suggested that females have a greater tendency to adopt maladaptive schemas than males, which may lead to a higher risk of eating disorders (Molina et al., 2023). Females typically show higher levels on perfectionistic schemas (e.g., Pursuing a perfect ideal body image or following strict dietary rules) (Deas et al., 2010; Jackson and Vares, 2013). Besides, females may more easily internalize other-directedness and rejection schemas. It means that compared to males, females have more negative self-perception, more concern of negative evaluation from others and may priorities the approval of others over their own personal preferences and needs (Sarin and Abela, 2003; Molina et al., 2023). Therefore, females may strive to adhere more closely to societal expectations which consequently increases their susceptibility to developing eating disorders.

In recent years, sociocultural factors have received increasing attention as a crucial influence on the gender difference of binge eating behavior. Cross-cultural research suggests that individuals, under the influence of family, peers, and media, internalize societal beauty standards. The perfect figure of idols always reflects gender differences in ideal body image. Male idols are expected to be thin but muscular (Pope et al., 1999), while female idol always receives an expectation of being ‘thin and light’ in body shape and weight (Jackson et al., 2021; Monocello, 2023). Under these aesthetic tendencies, men experience their muscles as ornamental, while women are dissatisfied with their normal body and always aspire to be thinner and slimmer (Hoodbhoy et al., 2015; Jackson et al., 2021; Kim and Han, 2021). For example, the ‘A4 challenge,’ which means a person can completely hide their waist with a width of 8.27′′, has become a social media expression of a slim body. Young women are more likely than young men to be interested in and take up this challenge (Jackson et al., 2021). It means that men and women are both subject to socio-cultural pressures, but they face different standards of the ideal body image (Jackson and Vares, 2013). As a result, men and women use different strategies to achieve their ideal body image. Women may control their eating behavior to achieve an ideal body image, whereas men may engage in physical exercise to build muscle (McCabe and Ricciardelli, 2003; McCabe and Ricciardelli, 2005). Excessive dieting is an important trigger of binge eating behavior (Elran-Barak et al., 2015; Chen et al., 2020). Therefore, women may be at higher risk of binge eating.

In fact, Chinese culture’s impact on gender differences in binge eating goes beyond gender differences in ideal body image. In the Chinese cultural context, women are often expected to conform to an ideal body image and appearance, while intelligence and achievement have always been associated with men. For example, when expressing that a couple is well-matched, the Chinese idiom “郎才女貌” is often used. In this idiom, “郎” refers to a man, “才” signifies talent or ability, “女” refers to a woman, and “貌” represents appearance. This idiom is used to describe a situation where the man possesses remarkable talent and the woman possesses a beautiful appearance, making them an ideal and harmonious couple. It seems that Chinese culture places more emphasis on the appearance of women than men. Thus, women in Chinese culture have higher standards regarding appearance and body image, which may lead to a higher risk of body dissatisfaction. Body dissatisfaction refers to a negative emotional state resulting from the discrepancy between one’s current body image and the idealized body state (Gao et al., 2019; Liu et al., 2019). Existing research has demonstrated that in China, female adolescents are more susceptible to distress from appearance evaluations and teasing, all of these may lead them have higher body dissatisfaction (Menzel et al., 2010; Chen et al., 2012). Our previous research indicated that body dissatisfaction contributes to binge eating behavior (Yan et al., 2022). Therefore, we propose that body dissatisfaction mediates the relationship between gender and binge eating behavior in Chinese culture (H1).

Self-acceptance refers to individual accepting all aspects of themselves (Ryff and Singer, 1996), including psychological and physical attributes (Meireles et al., 2021), and it usually results from an appropriate self-evaluation (Carson and Langer, 2006). The body is a part of the self. People with positive self-evaluations of their bodies have a correct self-knowledge; conversely, people who have negative body image tend to have negative self-evaluations, the latter leads to lower levels of self-acceptance. Therefore, it seems that body dissatisfaction may decrease self-acceptance, which was supported by the results of previous studies. For example, men who perceive themselves as overweight have lower levels of self-acceptance in the United States (Tager et al., 2006); body dissatisfaction is negatively correlated with self-acceptance (Cai et al., 2021; Romano et al., 2021; Zhao et al., 2023). On the other hand, not only has research shown a negative relationship between self-acceptance and binge eating (Tomba et al., 2014), but self-acceptance is often cited as an important factor in assessments of binge eating recovery (Fairburn et al., 2003; de Vos et al., 2017). Based on these evidences, we hypothesized that body dissatisfaction affect binge eating behavior through self-acceptance. That is to say, body dissatisfaction and self-acceptance might serve as a serial mediation between gender and binge eating behavior (H2).

**Figure 1:**
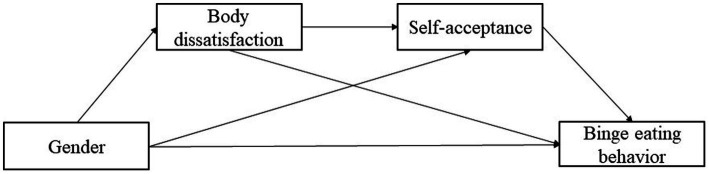
The serial mediation model hypothesized in the present study.

To sum up, the present study aims to understand the underlying psychological mechanism for gender differences of binge eating behavior in the Chinese culture. We suppose that Chinese women might pay more attention to their body image than men, which may lead to a higher risk of body dissatisfaction. Then the latter lower women’s self-acceptance, which finally result in more binge eating behavior. The research model is illustrated in Figure 1, and we intend to examine the following hypotheses:

*H1*: Body dissatisfaction mediates the relationship between gender and binge eating behavior.

*H2*: Body dissatisfaction and self-acceptance serve as a serial mediation between gender and binge eating behavior.

## Materials and methods

### Participants and procedures

The data were collected in June 2021. We recruited a sample of 715 university students from six randomly selected universities in China. Participants conducted pencil and paper questionnaire surveys in classroom, and received a small snack as compensation upon completion. They do not know the specific compensation until they receive it. All of them voluntarily participated in this survey and signed an informed consent form before completing the questionnaire. To ensure data quality, the exclusion criteria was: (1) The answer of equal or greater than half the length of the total scale are same (Curran, 2016); (2) Those who are non-undergraduate students. Finally, 703 students (227 males and 476 females) aged 18–25 were included in data analyses. The research was approved by the Ethics Committee of Guizhou Medical University. (Specific demographics are shown in Table 1).

**Table 1:** Descriptive statistics.

Variable		*N*	(%)	Variable	*M*	SD
Gender	Male	227	32.3	Age	19.89	1.18
	Female	476	67.7			
Grade	Freshman year	475	67.6			
	Sophomore year	140	19.9			
	Junior year	88	12.5			
Birthplace	City	145	20.6			
	Country	558	79.4			

Male = 1, Female = 2, Freshman year = 1, Sophomore year = 2, Junior year = 3, City = 1, Country = 2.

### Measures

#### Body dissatisfaction

The scale I in self-rating scale of aesthetic mental state (Zhou et al., 2000) was used to assess individuals’ level of satisfaction with their own body image (e.g.: ‘I was always afraid of being ugly and being judged ugly’). It is a 5-item self-report, using a 5-Likert point scale. The total score of all items is used as an indicator of body dissatisfaction. In the current study, Cronbach’s α was 0.796.

#### Self-acceptance

The self-acceptance subscale of Self-Acceptance questionnaire (SAQ) (Cong and Gao, 1999) was employed to assess individuals’ acceptance of reality (e.g.: ‘I’m always worried about being criticized and blamed by others’). It consists of 8 items, using a 4-point Likert scale. The total score of each participant reflects their level of self-acceptance, with higher scores indicating higher levels of self-acceptance. In the present study, Cronbach’s α was 0.825.

#### Binge eating behavior

Binge eating behaviors were assessed using the Chinese version of the Binge Eating Scale (BES) revised in 2014 (Gormally et al., 1982; Jin-Bo et al., 2014). The scale comprises 16 items, with a total score ranging from 0 to 46. A higher score indicates a more severe level of binge eating behavior. In our study, Cronbach’s α was 0.850.

### Statistical analysis

Data were analyzed with the Statistical Package for the Social Sciences for Windows, Version 26.0, according to Hayes described in his book (Hayes, 2017). Descriptive statistics and correlation analyses were conducted on all variables. Then according to Hayes, Model 4 in PROCESS v3.3 was used to test the mediation of body dissatisfaction, and Model 6 was used to test the serial mediation of body dissatisfaction and self-acceptance.

## Results

### Testing for common method bias

To address the issue of common method bias, a Harman’s single-factor test was conducted, incorporating all items from the four variables. The results indicated that the first factor accounted for 24.96% of the total variance, which fell below the 40% threshold proposed by Podsakoff et al. (2003). Consequently, the likelihood of significant common method bias confounding the interpretation of the data analysis is minimal.

### Descriptive statistics and correlation analyses

Table 2 displays the descriptive statistics and correlations among variables in the present study. Date revealed gender was positively associated with body dissatisfaction (*r* = 0.12, *p* < 0.01), binge eating behavior (*r* = 0.15, *p* < 0.01). It means that the score of body dissatisfaction and binge eating behavior are higher in females than males. Meanwhile, gender was negatively associated with self-acceptance (*r* = −0.11, *p* < 0.01). It means that the score of self-acceptance is lower in females than males. Body dissatisfaction was negatively associated with self-acceptance (*r* = −0.46, *p* < 0.01), while positively associated with binge eating behavior (*r* = 0.41, *p* < 0.05). In addition, there had significant negative correlations between self-acceptance and binge eating behavior (*r* = −0.34, *p* < 0.01).

**Table 2:** Descriptive statistics and correlation analyses of variables.

	*M*	SD	1	2	3	4
1 Gender			1			
2 BD	3.52	2.58	0.12^**^	1		
3 SA	19.59	3.44	−0.11^**^	−0.46^**^	1	
4 BEB	9.05	7.20	0.15^**^	0.41^**^	−0.34^**^	1

*N* = 703. BD, body dissatisfaction; SA, self-acceptance; BEB, binge eating behavior. Male = 1, Female = 2. *p < 0.05; **p < 0.01.

### Testing for mediation effect

After Chinese college students enter university, the supervision from parents and teachers during high school decreases sharply. They begin to arrange their own study and life independently, and they quickly socialize and mature (Geng et al., 2018). Therefore, the psychology and behavior of college students vary greatly with grade. On the other hand, the birthplace can seriously affect the self-esteem or self-acceptance (Weisskirch, 2007; Gonzalez-Guarda et al., 2013). Besides, subjects were not evenly distributed in terms of age. Therefore, we selected these three potential confounding factors as covariates in the data analysis. In fact, our own data in the present study does find that these variables affect the main variables we focus on. We use independent samples T-test /ANOVA analysis the effect of these sociodemographic factors on other variables. The results revealed that birthplace significantly affects self-acceptance (*p* < 0.05), and grade has a significant effect body dissatisfaction (*F* = 3.10, *p* < 0.05) as well as self-acceptance (*F* = 3.51, *p* < 0.05).

We used Model 4 in PROCESS v3.3 to test hypothesis 1, with age, grade, and birthplace as covariates (Hayes, 2017). The specifications are shown in Table 3. Gender was significantly associated with binge eating behavior (*β* = 0.19, *p* < 0.01. see Model 2 of Table 3) and body dissatisfaction (*β* = 0.25, *p* < 0.01. see Model 1 of Table 3). And the relationship between body dissatisfaction and binge eating behavior was also significant (*β* = 0.42, *p* < 0.001. see Model 2 of Table 3). The indirect effect reached a significance level, since CI of the above indirect effect did not include the zero value (as shown in Table 3). Therefore, H1 is supported. Body dissatisfaction partly mediated the relationship between body dissatisfaction and binge eating behavior.

**Table 3:** Regression coefficients, standard errors, and model summary information for the mediation effect of body dissatisfaction on binge eating behavior.

	Model 1 (BD)				Model 2 (BEB)			
	*β*	*t*	*SE*	95%CI	*β*	*t*	*SE*	95%CI
Constant	−1.07	−1.40	0.77	−2.58 ~ 0.43	−1.27	−1.83	0.69	−2.64 ~ 0.09
Age	0.04	1.02	0.04	−0.04 ~ 0.12	0.04	−0.98	0.04	−0.03 ~ 0.11
Grade	−0.15	−2.28^*^	0.07	−0.28 ~ −0.02	−0.01	−0.13	0.06	−0.13 ~ 0.11
Birthplace	0.03	−2.28	0.07	−0.15 ~ 0.22	0.11	1.24	0.09	−0.06 ~ 0.27
Gender	0.25	2.84^**^	0.08	0.09 ~ 0.41	0.19	2.66^**^	0.07	0.05 ~ 0.34
BD					0.42	12.28^***^	0.04	0.35 ~ 0.49
*R* ^2^	0.02				0.20			
*F*	3.80				34.40			

BD, Body dissatisfaction; BEB, Binge eating behavior. Male = 1, Female = 2. *p < 0.05, **p < 0.01, ***p < 0.001.

### Testing for serial mediation effect

**Figure 2:**
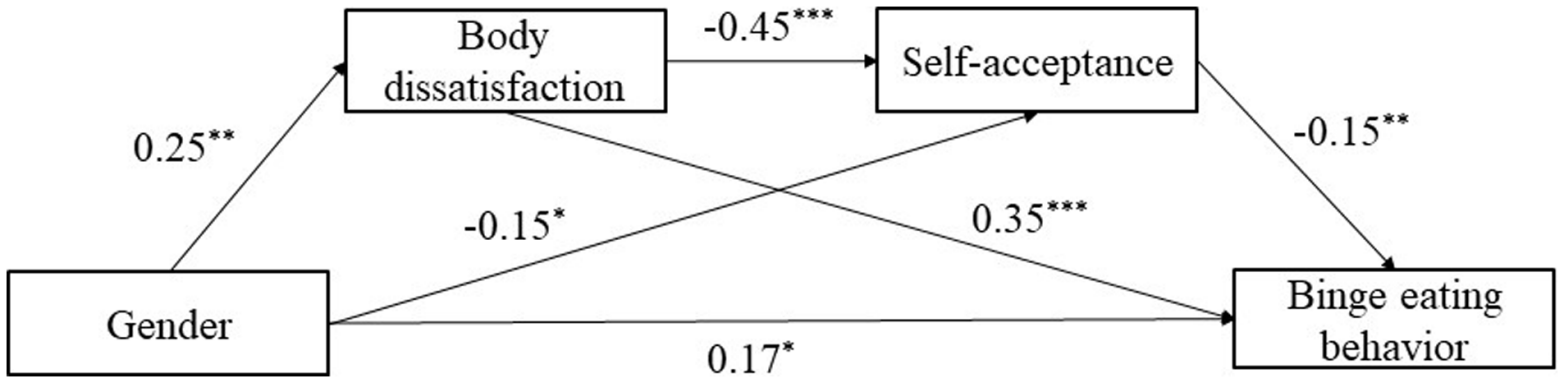
Theoretical research model with standard coefficients. Regression coefficients were obtained with age, grade, and birthplace in covariates in PROCESS Procedure for SPSS, where *** denotes that *p* < 0.001, ** means *p* < 0.01, * means *p* < 0.05, *N* = 703.

Gender, body dissatisfaction, self-acceptance, and binge eating behavior are all significantly associated, meeting the statistical requirements for mediation analysis of gender and binge eating behavior (Wen and Ye, 2014). With age, grade and birthplace as covariates, the mediating role of body dissatisfaction and self-acceptance in the relationship between gender and binge eating behavior was analyzed by using PROCESS v3.3 Model 6 in SPSS 26.0 compiled by Hayes (2017). The detailed results are presented in Table 4. Gender was a significant predictor of body dissatisfaction (*β* = 0.25, SE = 0.08, *p* < 0.01). Gender and body dissatisfaction are also significant predictors of self-acceptance (*β* = −0.15, SE = 0.07, *p* < 0.05; *β* = −0.45, SE = 0.03, *p* < 0.001). Finally, gender and body dissatisfaction, as well as self-acceptance, significantly predicted binge eating behavior (*β* = 0.17, SE = 0.07, *p* < 0.05; *β* = 0.35, SE = 0.04, *p* < 0.001; *β* = −0.15, SE = 0.04, *p* < 0.01), supporting H2. The results of the path coefficient test in the hypothetical model are shown in Figure 2.

**Table 4:** Regression coefficients, standard errors, and model summary information for the influence of gender in a model of binge eating behavior.

	Model 1 (BD)			Model 2 (SA)			Model 3 (BEB)		
	*β*	*t*	*SE*	*β*	*t*	*SE*	*β*	*t*	*SE*
Constant	−1.07	−1.40	0.76	1.95	2.87	0.68	−0.98	−1.41	0.69
Age	0.04	1.02	0.04	−0.08	−2.11^*^	0.03	0.03	0.75	0.03
Grade	−0.15	−2.28	0.07	0.11	1.83	0.06	0.01	0.14	0.06
Birthplace	0.03	0.34	0.09	−0.19	−2.32^*^	0.08	0.08	0.90	0.08
Gender	0.25	3.10^**^	0.08	−0.15	−2.15^*^	0.07	0.17	2.35^*^	0.07
BD				−0.45	−13.47^***^	0.03	0.35	9.25^***^	0.04
SA							−0.15	−3.98^**^	0.04
*R* ^2^	0.02			0.23			0.22		
*F*	3.80			42.52			31.91		

BD, body dissatisfaction; SA, self-acceptance; BEB, binge eating behavior. Male = 1, Female = 2. *p < 0.05; **p < 0.01; ***p < 0.001.

The mediating effect sizes of body dissatisfaction and self-acceptance in the relationship between gender and binge eating behavior are shown in Table 5. Body dissatisfaction (path 1) and self-acceptance (path 2) significantly mediated the effect of gender on binge eating behavior. Further, body dissatisfaction and self-acceptance serve as a serial mediation between gender and binge eating behavior (Path 3). The total effect value of gender on binge eating behavior was 0.30, and the direct effect value of gender on binge eating behavior was 0.17, and the total mediate effect accounted for 43.33% of the total effect. The mediating effect concludes three indirect effects: Path 1: Gender → Body dissatisfaction → Binge eating behavior (0.09), Path 2: Gender → Self-acceptance → Binge eating behavior (0.02), and Path 3: Gender → Body dissatisfaction → Self-acceptance → Binge eating behavior (0.02). The ratios of indirect effects of pathway 1, 2, and 3 to the total effect were 30, 6.67 and 6.67%. These three indirect effects reached a statistical significance level, since CIs of the above indirect effects did not include the value of zero.

**Table 5:** Direct and indirect effects of gender on binge eating behavior.

Path way	Estimate	SE	95% CI	Relative effect (%)
Total effect	0.30	0.08	0.14 ~ 0.46	
Direct effect	0.17	0.07	0.03 ~ 0.31	56.67
Total indirect effect	0.13	0.04	0.06 ~ 0.21	43.33
Gender → BD → BEB	0.09	0.03	0.03 ~ 0.15	30
Gender → SA → BEB	0.02	0.02	0.00 ~ 0.05	6.67
Gender → BD → SA → BEB	0.02	0.01	0.01 ~ 0.03	6.67

BD, body dissatisfaction; SA, self-acceptance; BEB, binge eating behavior. Male = 1, Female = 2.

## Discussion

The relationship between gender and binge eating behavior has been confirmed by numerous studies (Murnen and Smolak, 2015; Schaefer et al., 2019; Culbert et al., 2021). However, psychological mechanisms underlying the gender difference of binge eating behavior remain unclear. The present study further addressed this issue. Firstly, we examined the mediation effect of body dissatisfaction on the relationship between gender and binge eating behavior. Then we test a serial mediation role of body dissatisfaction and self-acceptance on the relationship between gender and binge eating behavior.

The powerful role of sociocultural factors in the development and maintenance of eating disorders has been documented in numerous studies (Jackson and Chen, 2007; Weissman, 2019). Today’s media and social culture, particularly Korean pop music TV programs popular in Asia countries, promote the idea that thinness is more attractive. In this context, body dissatisfaction becomes an important risk factor of eating disorders (Weissman, 2019; Yan et al., 2022). However, it is not clear that whether body dissatisfaction mediate gender difference in binge eating behavior.

Compared with boys, girls invest more in appearance-related activities such as photo editing (Mahon and Hevey, 2021), talking about weight with peers (Vincent and McCabe, 2000), and have higher desire to lose weight (Kashubeck-West et al., 2005). Compared with girls, boys are more active and acceptance. A qualitative research reported that boys could regard ideal image which posed on website in positive ways and have more confidence to improve their body image (Vincent and McCabe, 2000; Payne et al., 2011; Mahon and Hevey, 2021). It appears that boys might have a higher level of self-acceptance on body image, girls might have a higher level of body dissatisfaction. Many studies have supported this point (Vincent and McCabe, 2000; Ando and Osada, 2009; Jackson et al., 2021). On the other hand, the gender difference of body dissatisfaction might be further exacerbated in China. In Chinese culture, women are often expected to have ideal body image, while men are often expected to have high intelligence and achievement. This culture makes women pay more attention to body image, while men pay less attention to body image and more to talent. Our data confirm the gender difference in body dissatisfaction in China. Female’s body dissatisfaction is higher than that of male among Chinese college students.

Although boys are also subject to sociocultural pressures regarding their appearance (Xiaojing, 2017; Hicks et al., 2022), the details of such pressure are different between boys and girls. Girls are expected to be thin and slim whereas boys are often encouraged to be thin but muscular (Leit et al., 2001; Fischetti et al., 2020). Thus, men and women might use different strategies to achieve their ideal body image. Women may control their eating behavior to achieve an ideal body image, whereas men may engage in physical exercise to build muscle (Adrian et al., 2002; Pace Serena and Muzi, 2019). In China, dietary restraint is a very popular way to keep in shape in adolescent females (Jackson and Chen, 2014; Chen et al., 2020), and studies have shown that dietary restraint for too long can induce binge eating behavior (Elran-Barak et al., 2015; Chen et al., 2020). Thus, body dissatisfaction might play an important role on gender differences in eating disorders. Our data support this hypothesis, showing that body dissatisfaction mediated gender differences in binge eating behavior.

Body acceptance is often an important component of self-acceptance. Previous studies found that self-acceptance is negatively associated with negative body image (de Vos et al., 2017; Romano and Ebener, 2019). Consistently, our data show that self-acceptance is significantly negatively correlated with body dissatisfaction. In contrast, positive body image such as body appreciation increase self-acceptance (Levine and Smolak, 2016). More importantly, the improvement of self-acceptance frequently features as a crucial element in evaluating recovery from binge eating (Wilson, 1996; Fairburn et al., 2003). Reduction in binge eating was associated with increase in self-acceptance (Smith et al., 2006). Hence, it appears that body dissatisfaction mediates gender difference of binge eating behavior through self-acceptance. This hypothesis was supported by the results of present study. We found a serial mediation effect of body dissatisfaction and self-acceptance on gender difference of binge eating behavior. These results suggest that self-acceptance interventions targeting body image may help alleviate binge eating behaviors, but further research is needed.

This study has several contributions. At a theoretical level, it improves our understanding of the mechanisms of gender differences in binge eating behavior in a sociocultural perspective. In Chinese culture, body dissatisfaction and self-acceptance independently or through a serial way mediate the gender differences in binge eating behaviors. At a practical level, our results in the present suggested that self-acceptance interventions targeting body image may be an effective treatment for binge eating in women, although further experiments are still needed.

The present study has several limitations. First, the cross-sectional study design could not determine a causal relationship. Although significant differences were found in the data results, further longitudinal research is necessary to further confirm these conclusions. Second, body dissatisfaction, self-acceptance, and binge eating behavior were assessed by a self-report questionnaire, emphasizing the need for cautious interpretation of the research findings due to the possible biases that could arise from data analysis. Third, the convenience sampling employed in this study, which included only college students and had a gender imbalance with 73.1% female participants, highlights the need for future research to broaden the sample and address gender ratio disparities.

## Conclusion

Women’s body dissatisfaction is higher than men’s; while their self-acceptance is lower than that of men; body dissatisfaction and self-acceptance mediates the gender differences in binge eating behaviors independently. Further, they also mediate the gender differences in binge eating behaviors in a serial way. Therefore, special emphasis should be placed on the acceptance of body image in the prevention and intervention of binge eating disorder in China, especially among women.

## Data availability statement

The original contributions presented in the study are included in the article/supplementary material, further inquiries can be directed to the corresponding authors.

## Ethics statement

The studies involving humans were approved by the Ethics Committee of Guizhou Medical University. The studies were conducted in accordance with the local legislation and institutional requirements. The participants provided their written informed consent to participate in this study.

## Author contributions

CL: Conceptualization, Funding acquisition, Project administration, Supervision, Writing – original draft, Writing – review & editing. SL: Conceptualization, Formal analysis, Investigation, Writing – original draft. JY: Conceptualization, Formal analysis, Investigation, Methodology, Writing – original draft. XM: Conceptualization, Funding acquisition, Writing – review & editing, Supervision.
